# Sperm whale predator-prey interactions involve chasing and buzzing, but no acoustic stunning

**DOI:** 10.1038/srep28562

**Published:** 2016-06-24

**Authors:** A. Fais, M. Johnson, M. Wilson, N. Aguilar Soto, P. T. Madsen

**Affiliations:** 1 BIOECOMAC. Dept. of Animal Biology, La Laguna University, Spain; 2 Zoophysiology, Department of Bioscience, Aarhus University, Denmark; 3 Scottish Ocean Institute, University of St. Andrews, Scotland; 4 Institute of Biology, University of Southern Denmark, Denmark; 5 CREEM, University of St. Andrews, Scotland; 6 Murdoch University Cetacean Research Unit, Murdoch University, South Street, Murdoch, Western Australia 6150, Australia

## Abstract

The sperm whale carries a hypertrophied nose that generates powerful clicks for long-range echolocation. However, it remains a conundrum how this bizarrely shaped apex predator catches its prey. Several hypotheses have been advanced to propose both active and passive means to acquire prey, including acoustic debilitation of prey with very powerful clicks. Here we test these hypotheses by using sound and movement recording tags in a fine-scale study of buzz sequences to relate the acoustic behaviour of sperm whales with changes in acceleration in their head region during prey capture attempts. We show that in the terminal buzz phase, sperm whales reduce inter-click intervals and estimated source levels by 1–2 orders of magnitude. As a result, received levels at the prey are more than an order of magnitude below levels required for debilitation, precluding acoustic stunning to facilitate prey capture. Rather, buzzing involves high-frequency, low amplitude clicks well suited to provide high-resolution biosonar updates during the last stages of capture. The high temporal resolution helps to guide motor patterns during occasionally prolonged chases in which prey are eventually subdued with the aid of fast jaw movements and/or buccal suction as indicated by acceleration transients (jerks) near the end of buzzes.

“*It remains, then, to be inquired in what way the sperm whale (Physeter macrocephalus) usually does supply his enormous frame with sufficient food*” wrote the whaling surgeon Beale in 1840^1^. Today, almost 200 years later, that question is still relevant for the largest tooth-bearing predator on the planet, a major nutrient recycler in the world’s oceans^2^ responsible for an annual biomass turnover that has been compared to the combined catches of human fisheries^3^. Sperm whales are mainly teuthophagous predators that spend more than half of their life below 500 meters depth where they target meso- and benthopelagic prey. The main food source for sperm whales are medium sized squid (0.5 m and 1–3 kg), of which 70–80% are slow moving, ammoniacal species^4^,^5^, while the remainder are faster moving squid and fish targeted at meso- and epipelagic depths^6^,^7^,^8^. Occasionally, much larger squid such as jumbo, giant and colossal squid^4^,^9^,^10^ have been found in sperm whale stomachs, as well as rarer prey items ranging from sardines to seals^6^.

The body plan of sperm whales is dominated by a bluff-fronted head that, in old males, may constitute up to 1/3 of the body length^11^. Despite possessing a long, slender and underslung jaw with large conical teeth, there are in general no tooth marks on ingested prey items, and several examples of healthy sperm whales with severely deformed lower jaws led Berzin^6^ to conclude that “*there is reduction not only in the function of the teeth, but also that of the entire lower jaw in the digestive process of the sperm whale*”. In addition, there is a distance of several meters between the tip of the nose and the oropharyngeal opening, meaning that sperm whales must pass well over their prey before they can be ingested, leaving ample opportunity for escape. Thus sperm whales push a large nasal complex ahead of them while trying to capture small, often agile prey items with a narrow underslung jaw.

The apparent conflict between the gigantic nose of sperm whales and their foraging success^12^ has, in combination with their cryptic deep diving life style, led to a plethora of more or less informed speculations as to how sperm whales may go about subduing between 100 and 500 prey items per day^4^. Beale^1^ aired the common belief of whalers that sperm whales use the light coloration of their mouth region to attract prey. That theory was elaborated upon by Clark^13^ who proposed that foraging sperm whales hang motionless at depth by regulating their buoyancy via selective heating and cooling of the spermaceti organ. However, recently Amano and Yoshioko^14^ have shown that sperm whales are active predators that at times accelerate to speeds of more than 3 m/s during rapid changes in body orientation and sharp turns at depth^15^. Aoki *et al*.^16^ have further shown that such speed bursts are associated with squid ink and body parts passing cameras placed on the back of tagged sperm whales. These results show that sperm whales actively pursue prey during foraging dives. Hence, sperm whales are active predators, but the knowledge of how sperm whales find and subdue their prey is still sparse. Fristrup and Harbison^17^ suggested that sperm whales forage actively using vision to identify and locate prey. This visual predation hypothesis predicts that sperm whales may detect their prey by its bioluminescence or should consistently turn their ventral side towards the surface to facilitate stereo vision of silhouetted prey^6^.

In 1972, Norris and Harvey^18^ proposed that sperm whales use echolocation to find and discriminate prey by producing powerful biosonar clicks with their hypertrophied nasal complex. Recent experimental evidence has supported this theory by showing that the nasal complex is indeed a sound generator^19^,^20^,^21^,^22^ that can produce highly directional clicks with source levels (SLs) exceeding 235 dB re 1 μPa (peak-to-peak; pp) (equivalent to an energy flux density (EFD) of approximately 182 dB re 1 μPa^2^s), the highest instantaneous sound levels from any known biological sound source^23^. These source properties and the fact that sperm whales produce clicks throughout their foraging dives strongly suggest that sperm whales use long-range echolocation to find prey^7^,^24^,^25^,^26^,^27^. Gordon^24^ also proposed that echolocation is used during the last seconds of prey acquisition and compared the fast bursts of clicks (also called creaks) recorded from diving sperm whales with buzzes produced by bats in the terminal phase of prey capture^28^. Miller *et al*.^29^ went on to show that sperm whales display an increase in manoeuvring between some 12 s before and after the end of buzzes, with the peak of activity coinciding with the end of the buzzes. This coupling between buzzing and movements lends support to the hypothesis that sperm whale buzzes play a role in active prey acquisition as is the case for beaked whales^30^, porpoises^31^ and delphinids^32^. Furthermore, it was shown that buzzes occurred both while moving up and down in the water column^29^, suggesting that visual imaging of prey against down-welling light is not essential for sperm whale hunting as also evidenced by the findings of healthy, but blind sperm whales^6^.

However, while echolocation can explain how sperm whales find and track their prey, it does not address how the largest toothed whale is able to catch highly mobile nektonic prey which make up a portion of its diet. Norris and Møhl^12^ advanced the so-called biological big bang hypothesis suggesting that the powerful usual clicks of sperm whales serve to acoustically debilitate prey and thereby facilitate capture. This hypothesis presents a coherent scenario potentially explaining both the apparent hunting success of these large predators^7^ and the evolutionary driving force behind the hypertrophied nasal complex. A prerequisite for acoustic debilitation is that the prey is negatively affected by sound pressure levels within the capabilities of the sperm whale sound generator. Zagaeski^33^ exposed guppies to intense transients from a spark generator and found that 50% of the fish were stunned at received levels (RLs) of 235 dB re 1 μPa (pp). In an unpublished study from 1982, Norris and Møhl (B. Møhl pers comm) exposed the cephalopods *Loligo* and *Sepia* to exploding blasting caps and found that it took RLs of 253 to 266 dB re 1 μPa (pp) to debilitate these species. However, transients from both a spark generator and blasting caps have significant energy at low frequencies coupled with very fast rise times and therefore little spectral and temporal resemblance to toothed whale echolocation clicks. Indeed, when using ultrasonic transients closely mimicking those of toothed whale echolocation clicks on squid^34^ and on fish with swim-bladders^35^,^36^, RLs up to 226 dB re 1 μPa (pp) did not impair the swimming behaviour of either species. Hence, acoustic debilitation seems unlikely if sperm whales do not expose their prey to more than 235 dB re 1 μPa (pp) during prey capture. In fact source level estimates of buzz clicks from sperm whales have been estimated to be less than 210 dB re μPa (pp)^20^, so if sperm whale prey are indeed caught at the end of buzzes, their capture cannot be explained by acoustic debilitation.

A detailed understanding of how sperm whales catch prey and a specific test of the different and in some cases opposing hypotheses require fine scale data on the acoustic behaviour of foraging sperm whales and simultaneous information about the spatial relationship with their prey. Here we use high resolution sound and movement recording tags on free-ranging sperm whales to quantify their behaviour during prey captures. Our analysis supports previous reports that sperm whales forage actively and confirms that sperm whales do not consistently orient themselves as predicted to optimise the use of visual cues provided by the downwelling light. We also show that sperm whales do not debilitate prey acoustically, but rather employ a fast repetition rate buzz to provide high resolution echolocation sampling during active prey chases. We propose that prey are subdued near the end of the buzz with a rapid acceleration consistent with fast movements of the mandibles and the tongue to grasp and suck in prey.

## Results

### Foraging behaviour

**Figure 1:**
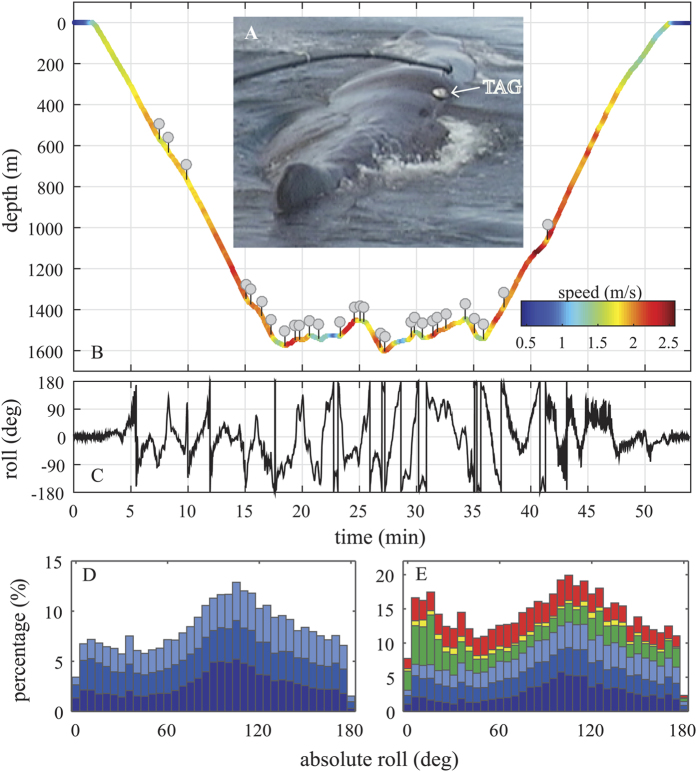
(**A**) Image of sperm whale 199b tagged in 2005 off northern Norway. The figure shows the tag position at the moment of deployment. (**B**) Section of a dive profile recorded from a male sperm whale off northern Norway. The colour indicates approximate swim speed estimated using a Kalman filter matching pitch angle to depth rate. This estimate loses accuracy at low pitch angles. Each grey circle marks a prey capture attempt (buzz). (**C**) Roll orientation of the diving sperm whale during the foraging dive. Distribution of the absolute roll angle during the foraging phase of pooled (**D**) shallow dives, depth <220 m (n = 31, 3 whales), and (**E**) deeper dives (n = 64, 6 whales). Coloured bars denote different animals.

During approximately 66 h of combined acoustic and movement data, the 6 tagged sperm whales performed a total of 82 foraging dives (Table 1). Echolocation periods, evidenced by production of usual clicks, lasted on average 38 (±7.6) minutes during deep foraging dives performed by the three whales tagged at low latitudes of the US East coast, amounting to 86% of dive duration. The three sperm whales off northern Norway echolocated on average 26 (±7.9) and 38 (±7.1) minutes during shallow and deep foraging dives, respectively, corresponding to 93% and 90% of dive duration. Buzzes were produced mostly during the bottom phase of foraging dives (Fig. 1, Table 1). Sperm whales foraging in the low-latitude habitat made on average 24 (±4) feeding buzzes per dive at depths ranging from 922 to 1197 meters. Whales foraging in the high latitude habitat switched between shallow (48–217 m) and deeper (253–1862 m) foraging layers. They performed fewer buzzes during shallow dives (4 ± 3) compared to deeper dives (15 ± 12). In total the whales produced 674 buzzes that lasted a median of 9.1 s (minimum: 2 s; lower quartile: 6.2 s; upper quartile: 14 s; maximum: 114 s). Of these, 567 buzzes matched the criteria used here to select independent buzzes (see Methods) and thus were included for further analysis (Table 1).

### Speed during buzzing

As exemplified in Fig. 1, tagged sperm whales moved forward at estimated speeds between 1 and 2.5 m/s. By design, the Kalman filter used to estimate speed in this figure tends to de-emphasize short bursts of high speeds associated with prey chases. Also, swimming speed is poorly estimated at low pitch angles. Therefore, the more robust method of orientation-corrected vertical speed was applied to 52 buzzes (n = 1 to 26 per whale), all with an absolute pitch angle greater than 45° throughout the buzz. This analysis shows that the whales moved forward during prey capture attempts at an average speed of 1.9 (±0.4) m/s (representing the mean over all buzzes of the average speed in each buzz), attaining minimum and maximum speeds of 0.6 m/s and 2.7 m/s (Table 1).

### Testing for multimodal sensing

Fristrup and Harbinson^17^ proposed that vision is essential for sperm whale foraging and hypothesized that whales should roll upside down to facilitate stereo vision^6^ of prey against down-welling light. To test this we computed the absolute roll angle of sperm whales throughout the foraging phase of dives, that is, between the first and last buzz within each dive^7^. Sections with high absolute pitch angles (>60°) were excluded from this analysis to avoid erroneous roll estimates due to gimbal lock^37^. The roll analysis was done separately for shallow dives (<220 m, n = 64, 3 whales) and deep dives (n = 31, 6 whales) to test for a preferential use of vision near the surface. The histograms of roll angles showed a bimodal distribution, with peaks around 10 and 110 degrees, for both dive types (Fig. 1C,D).

### Fast movements near the end of buzzing

**Figure 2:**
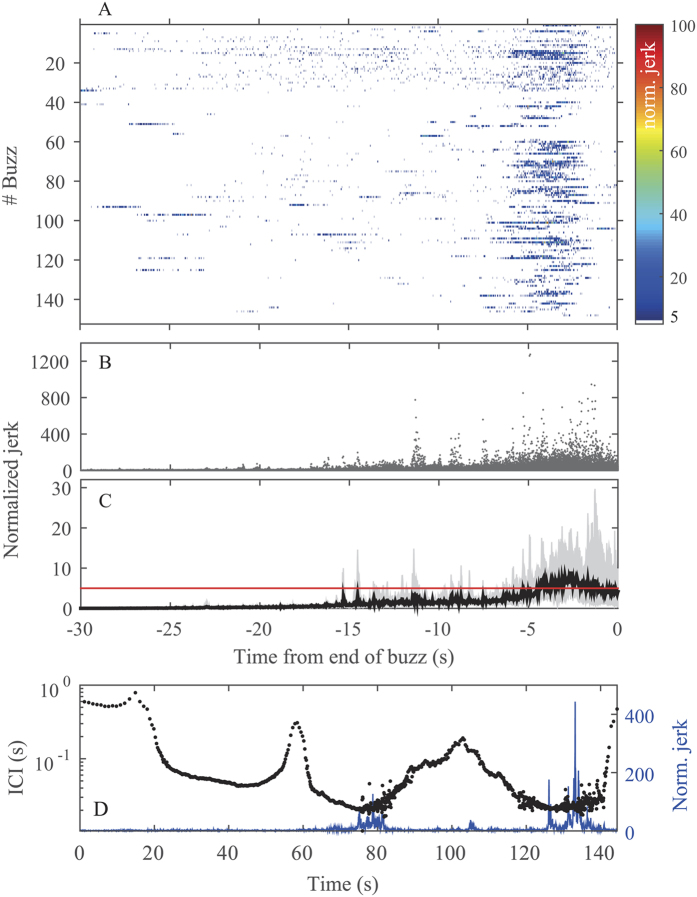
(**A**) Normalized jerk levels recorded in the 30 seconds prior to the end of each buzz (n = 154) of whale sw05_196a. Jerk was normalized to steady swimming. Values during steady swimming are represented in white. Values above the threshold set at 5, as indicated by the red line in (**C**), represent abnormally fast movements. (**B**) Normalized jerk relative to the end of buzz, pooling all buzzes from all whales with duration <21 s and time between consecutive buzzes (IBI) >16 s (n = 567) (see Methods for the definition of these thresholds). (**C**) Individual-weighted average of normalized jerk (black line) for buzzes shown in B. The upper and lower limits of the grey coloured area represent the maximum and minimum values of the individual averages, respectively. The red line denotes the 5 threshold used to detect a jerk transient. (**D**) Inter-click interval and normalized jerk signature of an echolocating sperm whale during a prey pursuit. The panel shows data from 10 seconds before the onset of the buzz and until 2 seconds after the end. Clicking alternates between faster and slower rates, accompanied by high jerk transients. The sperm whale may be tracking acoustically the moving prey by increasing its ICIs when prey escapes, and occasionally attempts to capture prey (as indicated by the high normalized jerk) when close enough.

The acceleration rate (jerk) was computed in the nominal approach phase and during the buzz to test for transients consistent with prey capture^38^. Jerk was normalized to the mean jerk during steady swimming (see Methods), and a normalized jerk threshold of 5, derived by the log-frequency distribution of the normalized jerk in approach and buzz phases, was used to detect strong transients. Normalized jerk transients above this threshold were found in 527 buzzes (93% of all buzzes analysed) and occurred in the last five seconds of buzzes in 70% of cases (Fig. 2). Peaks in normalized jerk during buzzes had a mean of 73 (interquartile range, 12 to 72), some 20 times higher than the normalized jerk during steady swimming (Table 1). Buzzes with normalized jerk transients and with high absolute pitch angle, for which accurate vertical speed estimation was possible, were included for further analysis (n = 27). For these 27 buzzes, the prey range at the start of each buzz was estimated by back-calculation, using the time delay between the normalized jerk peak to the start of the buzz to estimate the hand-off distance, i.e., the prey range at which whales radically alter the ICI at the onset of buzzes. The mean estimated hand-off distance for these buzzes was 9 m (interquartile range, 5 to 14 m).

### Acoustic output adjustment as a function of range to prey interception

**Figure 3:**
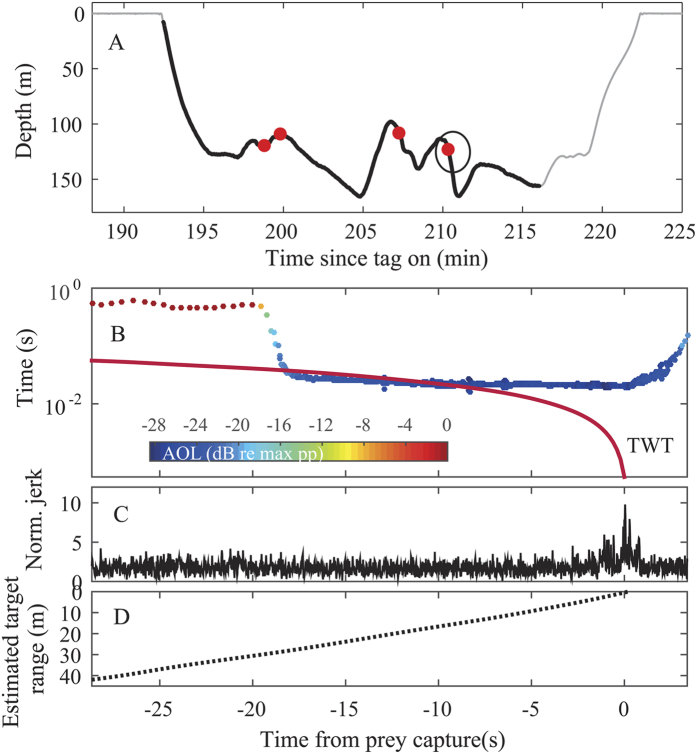
(**A**) Sperm whale shallow foraging dive profile with four prey capture attempts, i.e. buzzes, indicated by red circles. The black line indicates the echolocation phase of the dive. (**B–D**) represent different parameters from the start of the buzz to the peak in normalized jerk, taken as a proxy for prey interception. (**B**) A detailed view of the click rate during the final buzz (encircled in black in (**A**)). ICIs, from 10 s before the buzz to the end of the buzz, are colour coded by their relative AOL (dBpp re max pp). The red line marks the estimated two-way-travel time (TWT) from the sound source to the presumed prey capture location calculated from the forward speed of the whale. Note the log scale on the TWT axis. (**C**) Normalized jerk signature during the same period and (**D**) distance between the front of the whale and the presumed prey interception location, derived from the forward swimming speed of the whale, calculated using the orientation-corrected depth rate method.

Using the normalized jerk peaks as an indicator of when prey are intercepted during a buzz, we quantified the ICI and the apparent output level (AOL) as a function of time, and therefore range, prior to target interception (Fig. 3B–D) for steep buzzes in which speed estimation was reliable. Although ICI and AOL changed continuously through the approach and buzz, there was a major transition at the switchover from usual to buzz clicking. This was marked by an order of magnitude reduction in ICI from about 0.5 s to 20 ms (Fig. 3B). The impact of this on the potential acoustic field of view^39^ can be visualized by comparing the ICI with the estimated two-way travel time (TWT) from whale to prey (Fig. 3B). While the 0.5 s ICIs before the buzz leave a long lag time between the reception of an echo and the emission of the following click, the fast ICIs in the buzz are much closer to the TWT of the target.

**Figure 4:**
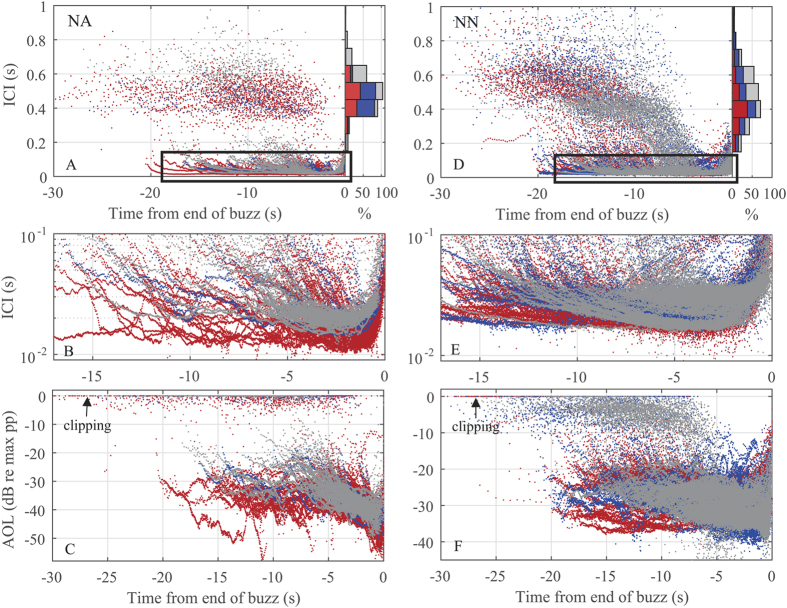
ICI’s of analysed buzzes as a function of time to the end of buzz for (**A**) sperm whales in the North Atlantic (NA; n = 154 buzzes), and (**D**) sperm whales in northern Norway (NN; n = 413 buzzes). The histograms on the right show the ICI distribution in the approach phase. Expanded view of the buzz phases in (**B**) North Atlantic and (**E**) northern Norway. Relative AOL (dBpp re max pp) as a function of time to the end of buzz for (**C**) North Atlantic sperm whales, and (**F**) sperm whales off northern Norway. Each colour represents a different animal.

During the usual click approach phase the tagged whales showed a bimodal pattern in ICI: the low-latitude whales produced stable ICIs up until the start of the buzz (Fig. 4A), whereas the sperm whales tagged off northern Norway tended to continuously decrease ICIs in this nominal approach phase (Fig. 4D). During buzzing all tagged whales continuously decreased ICI (Fig. 4B,E) with the exception of occasional fluctuations (e.g., Fig. 2D) which presumably track prey movements. As most usual clicks were clipped in the recording, an accurate measure of the AOL reduction from usual clicking to buzzing is not possible but a reduction of 1–2 orders of magnitude in peak pressure is conservative (i.e., a drop in level of 20–40 dB compared to the minimum outputs during the approach phase) (Figs 3B and 4). Within the buzz, where signal levels are well below clipping, there is a gradual decrease of AOL with time amounting to a roughly additional 10 dB level reduction over the course of the buzz (Fig. 4C,F).

### Sound exposures of prey

The sound exposure of prey to regular clicks was estimated by combining the median hand-off distance of 9 m with the maximum 235 dB re 1 μPa (pp) source level estimates of sperm whales (EFD: 182 dB re 1 μPa^2^s)^23^. Subtracting the approximately 20 dB of transmission loss over the 9 meters, the maximal sound exposure of prey just before the buzz will be no higher than some 215 dB re 1 μPa (pp) (EFD: 161 dB re 1 μPa^2^s). When sperm whales switch to a buzz they increase click rates and lower source levels by some 2 orders of magnitude^20^. The 20–40 dB reductions in source levels from the maximum of 235 dB re 1 μPa (pp) mean that prey exposures during the close encounters of the buzz are likely to be well below 200 dB re 1 μPa (pp) (EFD: 146 dB re 1 μPa^2^s).

**Figure 5:**
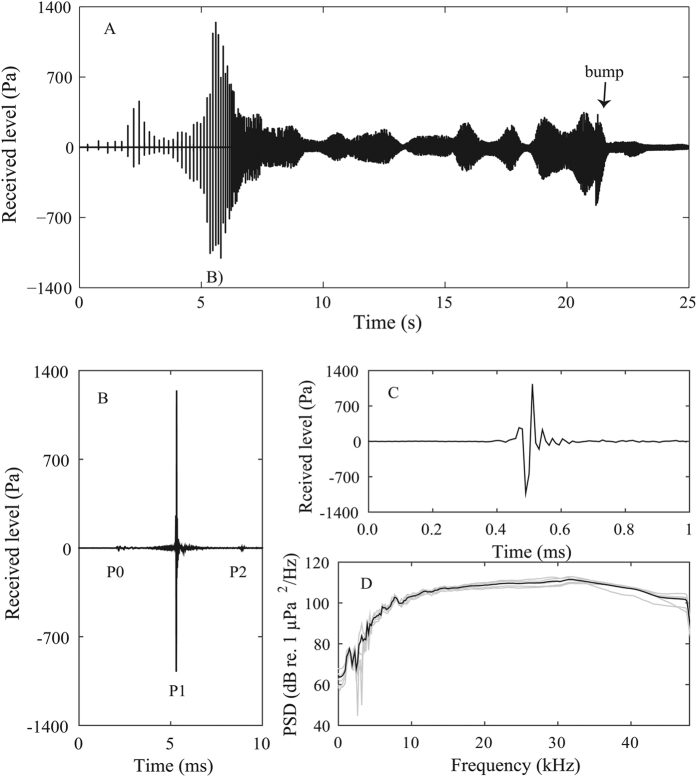
Data from a free-floating Dtag ensonified by a buzzing sperm whale that eventually bumps into the tag. (**A**) Absolute sound pressure level recorded by the tag. (**B**) The tag is ensonified at normal buzz rate clicking, and the clicks show the distinct monopulse nature of sperm whale on-axis clicks^21^, but the RLs never exceed 187 dB re 1 μPa (pp). (**C**) The waveform and (**D**) power spectral density (SPD) of the 5 most powerful clicks received on the tag (grey lines) and the mean overlaid (black line).

That estimate is supported by a fortuitous situation in one tag deployments where a Dtag after detachment from a whale at 630 meters depth was approached by the whale while buzzing. This ensonification provides a unique example of the sound field that may be received by a prey as it is approached by an echolocating sperm whale at depth (Fig. 5). The RLs of the buzz clicks in the tag varied as the whale scanned its directional sound beam across it, but never exceeded 187 dB re 1 μPa (pp) (EFD: 133 dB re 1 μPa^2^s) (Fig. 5). The received buzz clicks, which were not clipped due to the lower SL during buzzes, showed the distinctive monopulse nature of sperm whale on-axis clicks (Fig. 5B), with a duration of ca. 100 μs (Fig. 5C), a broad bandwidth and a centroid frequency >25 kHz (Fig. 5D). The 46 kHz recording bandwidth was clearly insufficient to sample the full frequency range of buzz clicks. The buzz followed the characteristic drop in ICI to 10–20 ms observed in other buzzes (Fig. 5A). The whale bumped into the tag 5 seconds before the end of the 20 s long buzz (Fig. 5A), consistent with the timing of jerk transients during buzzes recorded by tags on whales attempting to capture prey. In order to investigate the influence of the limited bandwidth of our recordings, we simulated a signal with a bandwidth of 96 kHz (sampling rate 192 kHz), using a spectrum that was close to identical to the spectrum of the most broadband signal in our recordings, but with the gentle high frequency roll off extrapolated so as to continue up to 96 kHz. We then evaluated the effect on the p-p amplitude measurements of this simulated signal by reducing the bandwidth again to 48 kHz, showing that the effect on the RL was below 1 dB.

## Discussion

In the last decades, the development of new tools to study the sounds and movements of free-ranging echolocating whales has extended our knowledge about sound production in sperm whales^21^,^22^,^40^,^41^, the acoustic properties of their clicks^23^,^42^,^43^, their echolocation behaviour^44^,^45^,^46^,^47^,^48^ and movements during foraging^14^,^15^,^16^,^29^,^49^. However, information about how the largest tooth-bearing predator in the world tracks and captures small and agile prey is still scarce. A number of hypotheses have been advanced, but they remain largely untested due to the difficulties in sampling fine-scale predator-prey interactions in the deep sea. To address this data gap we used multi-sensor tags to obtain a high-resolution picture of the movement and acoustic behaviour of free-ranging sperm whales during prey capture. This unique data set allows us to address the three hypotheses for how sperm whales hunt and capture their prey: the visual predation hypothesis^17^, the biological big bang hypothesis^12^ and the hypothesis of suction feeding^50^. To do so, we rely on a well-established indication of when whales are attempting to capture prey in the form of echolocation buzzes. Strong evidence for the role of buzzes in close prey approaches has been found in beaked whales where echoes from prey can be monitored^30^ and the consistent connection between buzzes and movement in sperm whales^29^, beaked whales^51^, porpoises^31^ and dolphins^32^ suggest that they serve the same role in other echolocating species. Using the end of the buzz as a first proxy for when whales have completed or abandoned the capture, we examine how tagged whales move in the preceding seconds. In the following we will use these new data to critically evaluate the three hypotheses for how sperm whales hunt and capture their prey.

### The visual predation hypothesis

Animals will likely make use of all sensory cues available to improve the efficiency of their activities^52^. Fristrup and Harbinson^17^ proposed that vision is central to sperm whale foraging and predicted a ventral-upward posture of sperm whales in order for them to use stereo-vision to search for silhouetted prey against a bright background. Such visual predation should be more prominent in well-lit shallower waters^12^. Our data show that tagged whales did not consistently swim upside down to spot and track prey silhouetted against the lit surface waters using stereo vision (Fig. 1B–D) augmenting the study by Miller *et al*.^29^ of downward and upward buzz directions. Instead, they spent a large portion of their foraging time rolled to one side which would enable only monocular vision of prey in down-welling light. Although sperm whales may be complementing echolocation with monocular vision, the fact that the whales use similar rolling behaviour in shallow and deep dives despite the very large difference in light levels, suggests that this behaviour has little to do with vision. Rather this finding may indicate that sperm whales have preferred approach angles for echo-guided prey capture. Specific body positions may favour the orientation of the whale’s sonar beam towards prey when approaching it, or may be linked to feeding mechanisms and/or to prey behaviour. Similar behaviour has been observed in some beaked whales, where specific approach angles and body orientations are adopted to capture particular prey^53^.

### Fast movements indicate strikes at prey

Many toothed whales use suction feeding to acquire prey^54^,^55^, and it has recently been shown that porpoises^31^, dolphins^32^ and beaked whales^30^ all subdue their prey by suction and/or raptorial feeding in the last part of the buzz. These feeding events require rapid movements in head and neck musculature which can be detected by accelerometers mounted near the head. As done in studies with non-echolocating marine mammals^37^,^56^, we used the differential of acceleration or jerk as a processing step to emphasise these sudden movements. Here we show that strong jerk transients are recorded consistently during buzzes by tags attached to the crest of the skull of sperm whales, and that they are seldom recorded outside of buzzes.

Caldwell *et al*.^50^ suggested that sperm whales ingest prey via a sucking motion of the tongue and gular region. That hypothesis has gained anatomical support by Werth^57^, who concluded that the morphology and relative positions of the tongue and the very large and flexible hyoid are indicative of suction feeding via an orifice formed by the oropharyngeal isthmus. In the current study, strong transients of jerk were found around 5 seconds before the end of the majority of buzzes (Fig. 2). As the differentiation used in calculating jerk de-emphasises relatively slow manoeuvres and swimming motions^37^,^58^, jerk transients are most likely an indication of rapid movements in the cranial and mouth region. Although acceleration signals associated with sound production have been shown recently in fin whales^59^, it is unlikely that the jerks found here are caused by clicks because jerk peaks occur late in the buzz, when acoustic output levels recorded by the tag are the lowest. Rather, the consistent presence of jerk transients near the end of buzzing, irrespective of buzz duration (Fig. 2A), strongly supports the interpretation that these are caused by rapid movements in the gular region during strikes at prey. We cannot infer from the jerk whether the strikes involve suction generation or raptorial jaw movements or both, although evidence of intact prey in stomach contents^6^ and the functional anatomy of the gular apparatus^57^ lend support to the former. Either way, if our interpretation is correct, the jerk transients do provide a strong indication of when prey are intercepted during the buzz phase

### Acoustic output adjustments to prey range

The transition from usual clicking to buzzing in the tagged sperm whales marks a dramatic gaze adjustment (Fig. 3B, refs 25 and 26). The tagged sperm whales reduced their ICIs by more than an order of magnitude when switching from usual to buzz clicking (Fig. 4A,B). Simultaneously they reduced the output level of their clicks by 20–40 dB (Fig. 4C,F). This echolocation behaviour is very similar to that described for smaller toothed whales, which increase click rates and reduce output levels in the terminal phase of prey capture^31^,^39^,^60^. A high click rate enhances the information flow on prey location to guide motor-patterns during close range manoeuvres, but at the potential expense of range ambiguity and spatial aliasing from distant targets^61^. A way to reduce the distance over which echoes can cause range ambiguity at high click rates, and thus to simplify the acoustic scene for perceptual organization, is to reduce the SLs. It is also possible that reduced SLs when clicking rapidly could relate to pneumatic restrictions of the sound generator of toothed whales^62^,^63^, but this does not alter the potential value of low SLs for close tracking of prey.

At a mean estimated distance of 9 meters to the prey, the tagged sperm whales switched from the approach to the buzz phase. This hand-off distance is about a body length of a female sperm whale and signifies the range from the prey to the sound source at the anterior part of the nasal complex, which is an additional 1–2 meters anterior of the oropharyngeal opening. A similar hand-off distance of 0.5 to 1 body lengths has been found in beaked whales^60^ and porpoises^31^. Hence, hand-off distances between the approach and buzz phase in echolocating toothed whales seem to be scaled with the size and possibly manoeuvrability of the predator^53^. Longer hand-off distances imply longer initial ICIs in buzzes, which is consistent with the overall click rate during buzzing that is about one order of magnitude slower in sperm whales compared to smaller toothed whales (Figs 3B and 4A–E; refs 31 and 61). This in turn implies less frequent updates of prey location in buzzing sperm whales. Given the similar forward speeds of different species of toothed whales during foraging (Fig. 3D; refs 14, 64 and 65), the clicking rate in buzzes may thus be more related to the absolute manoeuvrability of the echolocating whale^66^,^67^ than its speed^53^.

A hand-off distance of about 9 meters corresponds to a TWT to the targeted prey of 12 ms which is fairly close to the initial ICIs in buzzes (Fig. 3B). Our hand-off distance estimates are approximations as they assume that prey do not move when the buzz is initiated. When this assumption is not realized, it may result in an estimated TWT that is greater than the ICI as in the example of the 20-second long buzz in Fig. 3. It is more likely that sperm whales, as with beaked whales and bats, indeed use buzz ICIs that are greater than the TWT, but that the prey of the buzz in Fig. 3 starts to move away from the buzzing whales at some point during the buzz, prolonging the time from buzz start to terminal jerk at the end of the buzz. Pursuits of moving prey result in prolonged buzzes in both bats^68^ and toothed whales^8^. This could help explain the occasional long buzzes observed here, which can last up to two minutes being some 10 times longer than the normal short buzzes of sperm whales foraging in deeper waters (Fig. 2). In some of these longer buzzes, the clicking rate cycled several times between faster and slower modes, accompanied by strong jerk transients, suggesting that the whale is tracking the changing range to an elusive prey which it attempts to capture several times (Fig. 2D). Hence, sperm whales, like smaller toothed whales, target evasive prey that at times require long-distance pursuits (Fig. 2D^8^) and, occasionally, an increase in speed^14^,^15^,^16^ to be captured.

The ICIs of usual clicks during the approach phase were much longer than the estimated TWT at the start of buzzes (Fig. 4A,D), suggesting that sperm whales, like beaked whales, maintain a large sensory volume even after they have detected and selected a target. This likely facilitates tracking of several prey items for sequential capture^53^,^69^, supporting recent suggestions that sperm whales are able to organize perceptually a multi-target auditory scene^48^. The tagged sperm whales showed a bimodal clicking behaviour during the approach phase, where ICIs either remained stable (Fig. 4A) or displayed a gradual reduction (Fig. 4D) as prey were approached. Free-ranging dolphins have been shown to gradually decrease ICI as they close in on a target during the approach phase^70^. Conversely, beaked whales studied in the wild typically approach individual prey without such range locking in the approach phase^53^,^69^. The fact that sperm whales do not consistently conform to either of these modes suggests that clicking rate in the approach phase is possibly not species-specific, but could be dynamically adapted to different prey types or prey distribution found in specific habitats, which in turn may affect the echoic scene that must be processed by the echolocating toothed whales. Stable ICIs in the approach phase may be a way to deal with a complex auditory scene, calling for auditory stream segregation via slow sampling rates^69^. Conversely, if the whale targets a larger or more active prey in a simpler auditory scene, it may be able to apply range locking as it approaches it.

### Sound exposures of prey

The so-called biological big bang hypothesis predicts the use of acoustic debilitation to facilitate prey capture^12^. As found by Aoki *et al*.^15^ for sperm whales in the north Pacific, whales in our study occasionally swam at relatively high speeds during the foraging phase of dives. These bursts of speed were inferred in^15^ to be chases after active prey although the lack of acoustic data in that study meant that the connection between movement and echolocation phase could not be established. Acoustic data from the tags used in our study confirm that the fast swimming occurs just before and during buzzes and co-occurs with highly dynamic ICI patterns in buzzes. This strongly supports the interpretation that whales are chasing prey over distances of 10’s of meters which is inconsistent with prey debilitation that should involve effortless interception of immobile prey. Acoustic debilitation also implies that sperm whales should seek to maximize sound exposure of their prey. With a median hand-off distance of 9 meters from the approach to the buzz phase at which time the click outputs are reduced by two orders of magnitude, we show that sperm whales do not do that. Rather we estimate that prey items during the approach phase are unlikely to be exposed to more than 215 dB Re 1 μPa (pp), and that the low source levels in the buzz phase results in prey sound exposures well below 200 dB re μPa (pp) (Fig. 5). Those observations are also supported by a maximum received level of 187 dB re 1 μPa (pp) on a free-floating tag in front of a buzzing sperm whale (Fig. 5). Clearly this observation has to be evaluated with caution, given that it is derived from a single buzz from a single animal. Nevertheless, the back-calculated SLs from Fig. 5 and the 187 dB re 1 μPa (pp) exposure are consistent with independent buzz source level estimates from array recordings^20^. Furthermore, sperm whales have been reported to ingest a variety of non-food items^71^, and porpoises have been shown to use the same echolocation behaviour when targeting prey^31^ as when homing in on artificial targets^39^. These observations in concert lend weight to the notion that prey targeted by sperm whales are likely not exposed to more than 190 dB re 1 μPa (pp) during buzzing. These estimated RLs are at or below levels shown to have no effect on tested cephalopod or fish prey species with gas-bladders, which would likely be more prone to sound induced damage^34^,^35^,^36^.

Hence, it appears that sperm whales do not attempt to maximize the sound levels imping on their prey items. Instead they dramatically reduce source levels when they are within a body length of their prey resulting in exposure levels that are 1 to 2 orders of magnitude below those required to debilitate prey. The short, low-energy, broad-band and high-frequency clicks (Fig. 5) at high repetition rates in a buzz are therefore quite unsuited for prey debilitation, but rather well suited to provide both fast updates on prey location and to provide clutter reduction during the last few meters of the terminal prey capture phase as would be required to effectively track and capture evasive prey. In bats, buzz calls are of short duration and lower frequencies than search and approach calls, resulting in broad sound beams providing a wide field of view during close range target interception^72^. Interestingly, the higher centroid frequencies of sperm whale buzz clicks as compared to usual clicks would suggest an increase rather than decrease in directionality during buzzing, which is puzzling given the already high directionality of sperm whale usual clicks^23^. It may be that conformation changes in the sound producing nasal structures can offset the frequency effect by increasing the functional aperture, but clarification of this issue must await beam measurements of buzzing sperm whales.

## Conclusion

Here we have addressed several foraging hypotheses for sperm whales using high resolution on-animal measurements to uncover the movement and echolocation behaviour before and during prey capture. Sperm whales produce the most powerful biological sounds in the animal kingdom in order to echolocate prey at long ranges, but reduce acoustic outputs by several orders of magnitude when they are about their own body length from their prey. This behaviour is inconsistent with the big bang hypothesis indicating that the extreme nasal complex of sperm whales did not evolve to debilitate prey with sound, but rather to produce powerful clicks for long range echolocation of prey. There is likewise no indication that visual predation plays a significant role in sperm whale foraging. Instead, prey capture attempts by sperm whales are remarkably similar to those of other echolocating toothed whales comprising a sudden change in click rate and output level when the whales are about a body length from their prey. High repetition rate buzzes at low output levels provide high temporal and spatial resolution tracking to inform motor patterns in the last few meters before capture. While some prey targeted by sperm whales appear to be easy to subdue, others, often at shallower depths, require lengthy chases, leading to prolonged buzzes and higher than average swimming speeds. Strong and sudden changes in acceleration near the end of buzzes are supportive of the notion that sperm whales employ suction feeding to engulf their prey^57^ and continue buzzing during post-acquisition prey handling. Nonetheless, it still remains a conundrum how the sperm whale can keep track of a prey item during the final meters from when the prey drops below the sonar beam and until it reaches the buccal cavity, and we still do not understand the details of how the prey is engulfed. Hopefully, advances in animal-attached cameras will help answer these questions in the future.

## Materials and Methods

### Tagging

Field work was performed off the Eastern US seaboard (38°N, 70°W) where three sperm whales were tagged in July 2003, and in the Bleik canyon (69°25′N, 15°45′E), approximately 30 km W of Andøya, northern Norway, where three sperm whales were tagged in July 2005. Tagging was conducted under permits 369-1440-01, 981-1578 and 981-1707 from National Marine Fisheries Service to P. Tyack, and permit # 2005/7720-1 from the Norwegian Forsøksdyrsutvalg to P. T. Madsen. The study was performed in accordance with Woods Hole Oceanographic Institution, and as such, tagging methodology was approved by the Woods Hole Oceanographic Institution animal use and care committee.

Sperm whales tagged in the North Atlantic may have been adult females or immature males, both similar in size, whereas all three whales tagged off northern Norway were adult male sperm whales. Whales were tracked acoustically to predict their surfacing locations; once at the surface, whales were approached slowly from behind, and a Dtag (version 2) was placed using a 15 m cantilevered pole mounted on a rigid-hulled inflatable boat. All tags were placed in the neck region to ensure clear recordings of clicks and to maximize the chances of detecting acceleration from muscle movements associated with feeding (Fig. 1A). Tags were attached with a square array of 4 suction cups to minimise relative movement between the tag and the body. The Dtag is an archival device that samples 3-axis accelerometers, 3-axis magnetometers and pressure at 50 Hz^37^. Sound was recorded simultaneously with sensor sampling from one (2003) or two hydrophones (2005) at 96 kHz (16-bit). After the release of the tag from the whale, a VHF radio beacon in the Dtag aided tracking and recovery of the tag.

### Data analysis

Tag data were analysed using custom software in Matlab 7.5 (Mathworks). To exclude shallow submersions from further analysis, a foraging dive was defined as being deeper than 25 m and containing echolocation clicks. Foraging dives less than 220 m deep were considered shallow dives following Fais *et al*.^48^. Sound recordings were displayed as scrolling spectrograms (512 point FFT, Hann window, 50% overlap) to identify the start and end of echolocation clicking in each dive, along with the approximate time of buzzes. Clicks from tagged whales were separated from those of nearby clicking animals based on the consistently high RL of the former. Following Teloni *et al*.^8^, the precise start time of a buzz was defined as the first click in which the inter-click interval (ICI) dropped below 0.22 s. Conversely, the first click interval longer than 0.22 s was taken as indicating the end of a buzz. Within each dive, the time between the end of one buzz or buzz event and the start of the next was defined as the inter-buzz interval (IBI). Sequences of buzzes in close succession have been interpreted as capture attempts on the same target or a tight school of targets in other toothed whales^73^. Buzzes interrupted by air recycling pauses or by a brief increase in ICI (below buzz threshold) were, therefore, assigned as capture attempts of the same target and thus defined as a single buzz event, reaching durations up to two minutes (Fig. 2D). To avoid treating related buzz sequences as independent events, long buzz events (>21 seconds) comprised of different buzz sequences and buzzes spaced closely (<16 seconds apart) were excluded from further analyses. These criteria were chosen from log-frequency distribution plots of buzz length and IBI.

The emission time of each individual click was determined by a supervised click detector adjusted to the frequencies and ICIs previously reported for sperm whales^23^,^26^. The RL of the clicks at the tag, a proxy for the relative SL termed the apparent output level (AOL)^69^, was computed from the peak-to-peak amplitude expressed as dB re maximum pp^26^. In absence of information about when individual prey were detected and selected for capture, the approach phase to the prey^28^,^61^ was conservatively defined as the ten second time window preceding the transition into the buzz^53^.

Sensor data were decimated to 5 Hz and corrected for the tag orientation on the whale^37^. The orientation of the whale, represented by the Euler angles, pitch, roll and compass heading, was then estimated using standard procedures^37^. Swim speeds prior to, and during, buzzes were estimated from the depth rate and pitch angle of the whale *sensu* Miller *et al*.^74^). The accuracy of this orientation-corrected depth rate method decreases with declining absolute pitch angle and so the method was only used during buzzes in which the absolute pitch was consistently >45°. To visualize the approximate forward speed throughout the dive (Fig. 1), a two-state Kalman filter matching pitch angle to depth rate was used.

In order to detect changes in acceleration associated with prey capture and deglutition, the three accelerometer axes were differentiated and the norm of the resulting triaxial jerk was computed at each sample^38^,^51^,^58^. The maximum range of the accelerometer was ±2 g, and for this analysis sensor data were used at the full sampling rate of 50 Hz. In each dive the norm-jerk during buzzing was normalized by dividing by the RMS norm-jerk measured during seconds 10 to 40 of the descent phase, an interval that contained only steady swimming in all animals (normalized jerk coefficient [m/s^3^*(m/s^3^)^−1^]). The first 10 s of the descents were excluded to avoid strong fluke strokes. This normalization procedure compensates to some extent for different tag locations and was used for all jerk analyses. The normalized jerk signature within each buzz was inspected visually and the maximum peak in each buzz was identified. Assuming that the normalized jerk peak represents the moment of prey interception and so the time at which the whale-prey distance is zero, the target range as a function of time before the jerk peak was estimated for buzzes performed at high absolute pitch angles (>45 degrees) by integrating the estimated speed. This analysis assumes that the prey are stationary or move little in comparison with the whale during the approach. This may over-estimate the distance to prey that attempt to escape early in the buzz.

## Additional Information

**How to cite this article**: Fais, A. *et al*. Sperm whale predator-prey interactions involve chasing and buzzing, but no acoustic stunning. *Sci. Rep.* **6**, 28562; doi: 10.1038/srep28562 (2016).

## Figures and Tables

**Table 1:** Tagging information.

whale code	year	location	time recording	# foraging dives	# total/analysed buzzes	buzz depth [m]	buzz duration [s]	jerk [m/s^3^] #/buzzes	speed [m/s]* #/buzzes
201b	2003	NA	2 h	2	56/48	880 (222–1193)	8 (4–41)	41 (5–569)/48	1.3 (1.3–1.4)/3
202a	2003	NA	38′	1	17/16	787 (596–920)	5 (2–42)	59 (7–214)/16	1.8 (1.8–1.8)/1
206a	2003	NA	3 h 47′	5	103/89	852 (521–1024)	5 (2–58)	82 (7–945)/85	1.9 (1.3–2.2)/8
196a	2005	NN	24 h 17′	29	154/122	125 (87–492)	14 (7–114)	19 (6–174)/100	0.6 (0.6–0.6)/1
199a	2005	NN	20 h 42′	28	143/109	347 (30–1600)	12 (4–94)	47 (5–1275)/99	2 (1.8–2.2)/13
199b	2005	NN	16 h 46′	17	201/182	1343 (40–1854)	7 (4–25)	14 (5–196)/179	2.2 (1.2–2.7)/26

Whale codes are formed by the Julian day and the deployment order of the tag in that day. Foraging dives are defined as echolocation dives with a maximum depth greater than 25 m. Information is given as median (range). NA = North Atlantic; NN = northern Norway.

^*^Calculated using the orientation-corrected depth rate method for buzzes with absolute pitch >45°.
